# Polygon generation and video-to-video translation for time-series prediction

**DOI:** 10.1007/s10845-022-02003-1

**Published:** 2022-09-24

**Authors:** Mohamed Elhefnawy, Ahmed Ragab, Mohamed-Salah Ouali

**Affiliations:** 1 Department of Mathematics and Industrial Engineering, Polytechnique Montréal, 2500 Chemin de Polytechnique, Montréal, QC H3T 1J4 Canada; 2 CanmetENERGY, 1615 Lionel-Boulet Blvd., P.O. Box 4800, Varennes, QC J3X 1P7 Canada; 3 Faculty of Electronic Engineering, Menoufia University, Menouf, 32952 Egypt

**Keywords:** Time-series prediction, Polygon generation, Video-to-video translation, Generative adversarial network (GAN), Data streams, Deep learning (DL)

## Abstract

This paper proposes an innovative method for time-series prediction in energy-intensive industrial systems characterized by highly dynamic non-linear operations. The proposed method can capture the true distributions of the inputs and outputs of such systems and map these distributions using polygon generation and video-to-video translation techniques. More specifically, the time-series data are represented as polygon streams (videos), then the video-to-video translation is used to transform the input polygon streams into the output ones. This transformation is tuned based on a model trustworthiness metric for optimal video synthesis. Finally, an image processing procedure is used for mapping the output polygon streams back to time-series outputs. The proposed method is based on cycle-consistent generative adversarial networks as an unsupervised approach. This does not need the heavy involvement of the human expert who devotes much effort to labeling the complex industrial data. The performance of the proposed method was validated successfully using a challenging industrial dataset collected from a complex heat exchanger network in a Canadian pulp mill. The results obtained using the proposed method demonstrate better performance than other comparable time-series prediction models. This allows process operators to accurately monitor process key performance indicators (KPIs) and to achieve a more energy-efficient operation.

## Introduction

Climate change is one of the most important challenges that is urgent to be tackled due to its dangerous effects on different natural aspects (Environment challenges | Climate Action, 2021). Increase of greenhouse gas (GHG) emissions in the atmosphere is one of the main reasons for this climate change challenge worldwide. The use of fossil fuels in heavy process industries have primarily led to such emissions. These industries are the largest energy-consuming sectors of the industry’s total delivered energy (Li & Tao, 2017). In Canada, GHG emissions increased form 600 mega tonnes of carbon dioxide equivalent (Mt CO_2_ eq.) in 1990 to 730 Mt CO_2_ eq. in 2019 (increase by 21.4%) (National Inventory Report, 2019). According to that report, oil, and gas industry (26%) and transport (25%) are the primary causes of such growth of Canada’s emissions. Among the reasons for the GHG emissions and excessive energy consumption of such industries are the inefficient monitoring and control of such complex and highly dynamic processes. In such processes, a set of key performance indicators (KPIs) are used for monitoring their health state.

Inefficient control of these KPIs results in various environmental and economic impacts in terms of harmful emissions, excessive maintenance, and unexpected downtime (Andersson & Thollander, 2019). Therefore, developing an accurate prediction model for these KPIs is an urgent need for the sake of accurate KPIs monitoring and optimization that help maintain an energy-efficient operation and mitigate such environmental impacts and economic losses (Rolnick et al., 2019).

Most of heavy industrial systems are characterized by highly nonlinear and dynamic operation which make monitoring and prediction of their KPIs more challenging. These nonlinear processes are hard to model and predict their unexpected responses based on the expert knowledge alone (Ragab et al., 2016; Ragab et al., 2019b). The system response is continuously changing using the same inputs at different time instants. Moreover, the superposition principle can not apply, and therefore dealing with multiple input variables is a tedious task. Fortunately, these industrial systems are equipped with numerous number of sensors that acquire huge amount of data of different types. One of the major data sources available in such industrial systems is the time-series data. This time-series data acts as an important opportunity to build accurate data-driven models using machine learning (ML) techniques. Data-driven modeling shows promising solution compared to classical analytical techniques such as autoregressive integrated moving average (ARIMA), simple exponential smoothing (SES), Holt Winter’s exponential smoothing (HWES) (Box et al., 2015; Brown & Meyer, 1961; Kedem & Fokianos, 2005; Pan, 2010) which are not effective in case of highly dynamic complex systems with several interacted components. However, most of machine learning techniques such as artificial neural network (ANN), decision trees and support vector regression (SVR) used for time-series prediction in the industry made assumptions and can not capture the actual distribution of data of such non-stationary dynamic processes (Alpaydin, 2010; Franklin, 2005; Lapedes & Farber, 1987). This may result in inaccurate performance of these models and hinder their deployment in such cases.

Another challenge in processing the industrial data is the labeling phase. Correct labeling of the industrial data in alignment with the input variables is an indispensable need for training and testing various data-driven modeling techniques. Unfortunately, labeling of this type of data is a tedious process that heavily involves the human experience which is rare (Ragab et al., 2019a). Even though with existence of such expertise, the labeling process may not be done in an appropriate way that leads to inefficient model building. Fortunately, the deep learning (DL) approach offers an opportunity to tackle the above-mentioned limitations. It has been proven that DL achieves better predictive performance compared to other classical ML predictors (LeCun et al., 2015; Lv et al., 2016; Goodfellow & Bengio, 2017).

For accurate time-series prediction, there is a need to learn a mapping function that converts the input time-series variables into the targeted outputs (KPIs in the industrial context). In other words, a data distribution matching problem needs to be solved aiming to train a model such that the conditional distribution of the predicted KPIs given the input variables resembles that of real KPIs. Conditional generative modeling can be a promising approach for solving this type of problems (M. Y. Liu et al., 2021). One of the state-of-art DL conditional generative modeling techniques is the conditional generative adversarial networks (cGANs) (Goodfellow et al., 2014; Isola et al., 2017) that are used for data augmentation, mapping of images or videos from one domain to another, creating image filters and others.

The distribution matching can be facilitated through a better data representation (Schat et al., 2020). In fact, data representation pathway is the optimal approach for practitioners and researchers in the DL field. These researchers are still developing new architectures and/or optimizing the existing ones without looking over the available data and maximize their value before exploitation. The available data acts as a fuel for training the DL architectures. Accordingly, focusing on improving the data representation is an urgent need for better modeling performance. Data-centric AI (Andrew Ng Launches A Campaign For Data-Centric AI, 2021; Wu, 2021) is an emerging approach nowadays for improving the quality of data used for training DL models. Researchers and practitioners are recently starting to organize several occasions with the goal of obtaining the best data representation that achieve the highest prediction performance using the same DL architecture. The Data-Centric AI Competition Hackathon is one of these occasions (Data-Centric AI Competition, 2021).

To overcome the above mentioned limitations, fill the gaps and exploit the opportunities of the generative modeling power of cGANs and improve data representation using data-centric AI approaches, this work adopts the polygon generation (PG) technique proposed in Elhefnawy et al. (2021) to transform the time-series data into polygon streams (videos). These videos represent all interrelationships between the time-series inputs and their change over time using Hamiltonian cycles. For mapping the polygon streams of the input variables into that of the KPIs (outputs), we propose to use the video-to-video translation (3D-CycleGAN) technique introduced in Bashkirova et al. (2018). This technique is based on the cycle-consistent generative adversarial networks as an unsupervised method in which the data does not need to be paired, accordingly, it saves the effort of the labeling process done by the process expert. A model trustworthiness metric is used for tuning the 3D-CycleGAN to ensure the consistency of the acquired polygon streams with the original polygon streams. After obtaining the translated polygon streams representing the predicted KPIs, an image processing procedure is applied for every video frame to recover the numerical values of the KPIs. In industrial context, the historical data streams are used to train the unsupervised video-to-video translation architecture. This trained architecture is then used to predict the system’s KPIs given the unseen input data streams. The main contributions of this work are summarized as follows:

- The proposed method makes use of both the powerful representation of the PG technique and the breakthrough of the deep generative modeling. By combining these two approaches, an accurate and robust KPI prediction technique is developed by representing the time-series data as polygon streams using the PG technique, then efficiently mapping these polygons (inputs) into numerical values (outputs) through the unsupervised cGAN. This aims at maximizing the knowledge extracted from the challenging industrial time-series data.
- This proposed method is validated using an energy-intensive concentrator equipment in a pulp & paper mill located in Canada and the results show that it outperformed other common DL time-series predictors. The method accurately predicts three important KPIs in the concentrator: the evaporated water, the concentrator efficiency, and the fouling index. This helps maintain an energy-efficient operation and helps mill’s operators mitigate environmental impacts and economic losses.

The rest of this paper is organized as follows. “Background & related work” section provides a background on polygon generation for time-series data and unsupervised video-to-video translation (3D-CycleGAN), in addition to some related work on DL time-series prediction in industrial systems. “Proposed method for time-series prediction” section presents the proposed method with its detailed steps. “Case study: concentrator in heat recovery network (HRN)” section shows the industrial case study: the concentrator equipment used to validate the proposed method and the experimental setup. “Results, discussion & future work” section discusses the results and gives insights and future work directions. Finally, “Conclusion” section concludes the paper.

## Background & related work

This section discusses the background and related work to time-series prediction in industrial systems using deep learning methods. It also presents the two main methods used in the proposed method to tackle the problem of time-series prediction. The first method is the polygon generation as an efficient data representation technique that converts the numeric time-series observations into polygon streams (videos). The second one is the video-to-video translation method that maps polygon videos of time-series inputs into outputs (KPIs).

### Deep learning for time-series prediction in industrial systems

The DL has become an opportunity for developing more accurate time-series predictive models in highly dynamic industrial systems compared to classical machine learning algorithms (Gamboa, 2017; Nadim et al., 2022; Zhao et al., 2020). Convolutional neural network (CNN) is one of the DL architectures that are used commonly in image, speech and time-series data (Borovykh et al., 2017; Huang et al., 2015; LeCun et al., 1995). The interested readers can find more applications on DL in time series prediction in the comprehensive review papers (Gamboa, 2017; Han et al., 2021). In what follows, some related work are presented.

A deep CNN combined with an adaptive time-series window (ATSW) is used in Hoermann et al. (2018) and validated using a time-series data collected from an industrial furnace. Another augmented multi-dimensional CNN is used in Hoermann et al. (2018) for industrial soft sensing. Recurrent DL architectures such as LSTM (Gers et al., 2000) has been used extensively in the literature for time-series prediction. A convolutional LSTM encoder-decoder architecture is proposed in Essien & Giannetti (2020) for smart manufacturing and validated using real data from an industrial plant in United Kingdom. A spatiotemporal attention-based LSTM is used in Yuan et al. (2021) for developing industrial soft sensor models. Besides, for quality prediction in manufacturing, LSTM is used in Bai et al. (2021) as a regression tool along with AdaBoost for model’s reinforcement. An LSTM architecture is used in Soualhi et al. (2021) in the pulp and paper industry using a dataset collected from a heat exchanger located in Canada. Another architecture close to that of LSTM is called gated recurrent unit (GRU) which has less number of gates (less parameters) and used in case of smaller datasets (Cho et al., 2014). A bidirectional GRU with weighted features averaging is used in Wang et al. (2019) for smart manufacturing.

However, most of the architectures used in the literature works in a supervised way with paired inputs and outputs for training. This pairing needs an additional effort by the process expert. In addition, it is hard to capture the true distribution of the complex industrial data in case of highly dynamic nonlinear process. Therefore, more focus is needed for better data representation for the maximal exploitation of the available industrial data.

### Polygon generation for data representation

A data representation technique called “Polygon Generation” was proposed in Elhefnawy et al. (2021) to map the numerical observations into polygon images. These polygon images are used for training a deep learning model for accurate classification. This technique was validated using a challenging dataset collected from a reboiler system in a pulp and paper mill located in Canada. Due to its effectiveness in representing the numerical data, we are motivated in this work to adopt that polygon generation technique for time-series prediction in highly dynamic industrial processes. More detailed steps on polygon generation are illustrated via an illustrative example in Elhefnawy et al. (2021). In what follows, we summarize these steps through another toy example.

**Fig. 1:**
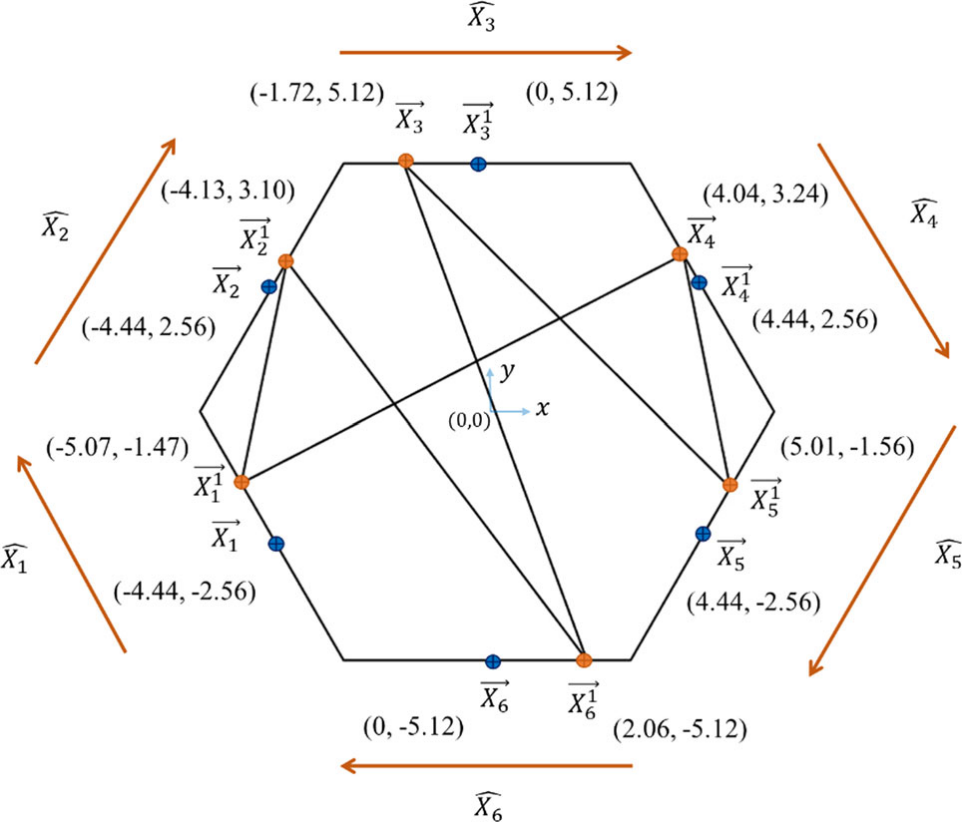
A polygon generated from a numeric observation of six data variables using the method proposed in Elhefnawy et al. (2021)*.* All variables are numbered in a clockwise direction

This numerical example comprises numerical data with six input variables and four outputs. Figure 1 shows a regular hexagon where each side represents an input variable. The point coordinates $\overrightarrow{{X}_{j}^{k}}$(in orange) that represent the standardized values of observation $k$ for the variable ${X}_{j}$ are calculated using Eqs. (1) and (2), where $j={1,2},\dots ,6$.

1 $${Z}_{kj} = \frac{{x}_{kj}-\overline{{X }_{j}}}{{\delta }_{j}}$$

2 $$\overrightarrow{{X}_{j}^{k}}=\overrightarrow{{X}_{j}}+\left({Z}_{kj}*\widehat{{X}_{j}}\right)$$

where ${x}_{kj}$ is the actual numeric value of observation $k$ for the variable ${X}_{j}$, $\overline{{X }_{j}}$ and ${\delta }_{j}$ are their mean value and standard deviation, respectively, ${Z}_{kj}$ is the standardized value of observation $k$ for the variable ${X}_{j}$, $\widehat{{X}_{j}}$ represents the unit vector of each polygon side and $\overrightarrow{{X}_{j}}$ are the point coordinates (in blue) that represent the zero standardized value of the variable ${X}_{j}$.

**Fig. 2:**
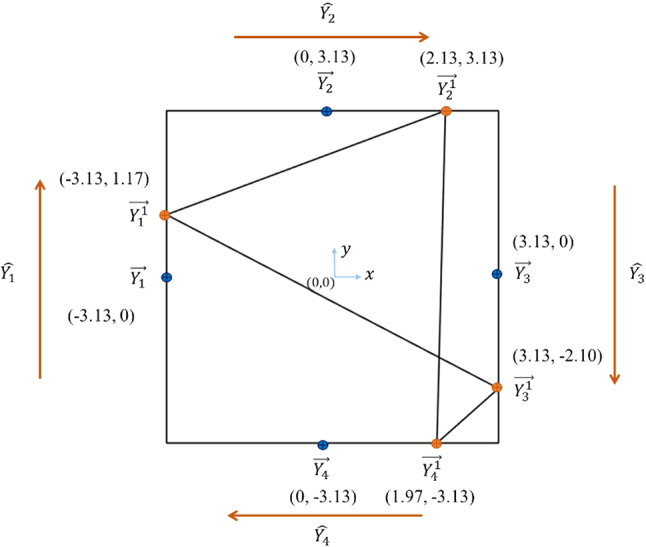
A polygon generated from a numeric observation of the four outputs. All outputs are numbered in clockwise direction

Table 1 shows the $\overrightarrow{{X}_{j}^{k}}$ values calculated using Eqs. (1) and (2). Similarly, this procedure is applied to the numerical outputs. Figure 2 shows a square that represents the four outputs, where each side represents the output ${Y}_{h}$, where $h=\mathrm{1,2},\mathrm{3,4}$. Table 2 shows the calculations of $\overrightarrow{{Y}_{h}^{k}}$ (in orange) that represent the standardized outputs ${Z}_{kh}$, where ${y}_{kh}$ is the numeric value of observation $k$ for the output ${Y}_{h}$, $\overline{{Y }_{h}}$ and ${\delta }_{h}$ are their mean value and standard deviation, respectively, $\overrightarrow{{Y}_{h}}$ are the point coordinates (in blue) that represent the zero standardized value of the output ${Y}_{h}$ and $\widehat{{Y}_{h}}$ represents the unit vector of each polygon side.

**Table 1:** Calculation of point coordinates $\overrightarrow{{X}_{j}^{1}}$ on the sides of the polygon for a numeric observation with six variables shown in Fig. 1, where $\widehat{q}$ and $\widehat{l}$ are the unit vectors of $x$ and $y$ directions, respectively

$j$	${x}_{1j}$	$\overline{{X }_{j}}$	${\delta }_{j}$	${Z}_{1j}$	$\widehat{{X}_{j}}$	$\overrightarrow{{X}_{j}}$	$\overrightarrow{{X}_{j}^{1}}$
1	69.55	68.54	0.8	1.26	$-0.5 \widehat{q}+0.87 \widehat{l}$	$-4.44 \widehat{q}-2.56 \widehat{l}$	$-5.07 \widehat{q}-1.47 \widehat{l}$
2	125.24	118.12	11.48	0.62	$0.5 \widehat{q}+0.87 \widehat{l}$	$-4.44 \widehat{q}+2.56 \widehat{l}$	$-4.13 \widehat{q}+3.10 \widehat{l}$
3	853.77	860.36	3.83	-1.72	$1 \widehat{q}+0 \widehat{l}$	$0 \widehat{q}+5.12 \widehat{l}$	$-1.72 \widehat{q}+5.12 \widehat{l}$
4	8.95	11.42	3.13	-0.79	$0.5 \widehat{q}-0.87 \widehat{l}$	$4.44 \widehat{q}+2.56 \widehat{l}$	$4.04 \widehat{q}+3.24 \widehat{l}$
5	4035.1	4120.38	74.16	-1.15	$-0.5 \widehat{q}-0.87 \widehat{l}$	$4.44 \widehat{q}-2.56 \widehat{l}$	$5.01 \widehat{q}-1.56 \widehat{l}$
6	59.94	45.33	7.09	-2.06	$-1 \widehat{q}+0 \widehat{l}$	$0 \widehat{q}-5.12 \widehat{l}$	$2.06 \widehat{q}-5.12 \widehat{l}$

**Table 2:** Calculation of point coordinates $\overrightarrow{{Y}_{h}^{1}}$ on polygon sides for a numeric observation with four outputs shown in Fig. 2, where $\widehat{q}$ and $\widehat{l}$ are the unit vectors of the $x$ and $y$ directions, respectively

$h$	${y}_{1h}$	$\overline{{Y }_{h}}$	${\delta }_{h}$	${Z}_{1h}$	$\widehat{{Y}_{h}}$	$\overrightarrow{{Y}_{h}}$	$\overrightarrow{{Y}_{h}^{1}}$
1	3.65	3.46	0.16	1.17	$0 \widehat{q}+1 \widehat{l}$	$-3.13 \widehat{q}+0 \widehat{l}$	$-3.13 \widehat{q}+1.17 \widehat{l}$
2	102.29	23.88	36.81	2.13	$1 \widehat{q}+0 \widehat{l}$	$0 \widehat{q}+3.13 \widehat{l}$	$2.13 \widehat{q}+3.13 \widehat{l}$
3	63.99	15.17	23.25	2.10	$0 \widehat{q}-1 \widehat{l}$	$3.13 \widehat{q}+0 \widehat{l}$	$3.13 \widehat{q}-2.10 \widehat{l}$
4	0.19	8.47	4.2	− 1.97	$-1 \widehat{q}+0 \widehat{l}$	$0 \widehat{q}-3.13 \widehat{l}$	$1.97 \widehat{q}-3.13 \widehat{l}$

Figures 1 and 2 shows one possible connection between the points on polygon sides representing the observation values for input variables and outputs, respectively. This polygon generation technique represents all interrelationships between variables and outputs through Hamiltonian cycles (Elhefnawy et al., 2021). Accordingly, each observation is represented as multiple images with all possible connections between points on polygon sides. The algorithm proposed in Hurley and Oldford (2010), Wegman (1990) is used for this multiple images’ generation step. More details are found in Elhefnawy et al. (2021).

### Unsupervised video-to-video translation

There are two main approaches in data-driven modeling; discriminative modeling and generative modeling (Ng & Jordan, 2002). Given input variables $X$ and outputs *Y*, the discriminative modeling predicts the probability distribution of outputs $Y$ given the variables $X$, denoted mathematically as $P(Y|X)$ whether in classification problems (categorical $Y$) or regression problems (continuous $Y$). The generative modeling on the other hand predicts the data distribution of the inputs $X$ given the outputs $Y$ ($P(X|Y)$) (Jebara, 2012).

**Fig. 3:**
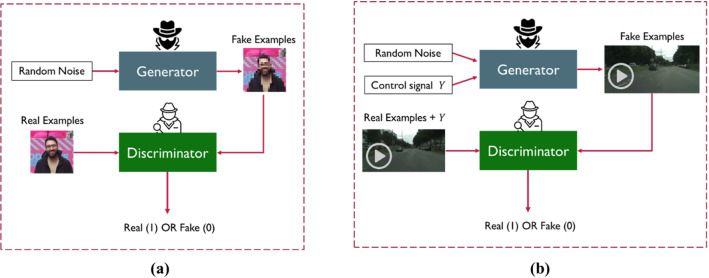
A simplified schematic of **a** unconditional GAN and **b** conditional GAN. Both unconditional and conditional GANs can be applied to several data types such as images, video, text, etc.

One of the state-of-art techniques for generative modeling is the generative adversarial networks (GANs). They are first introduced in Goodfellow et al. (2014). The GAN architecture has two main components (networks); generator and discriminator. The generator works on synthesizing some fake examples, acting as a forger who tries to mimic the real examples (images, text, videos, etc.) (See Fig. 3). The discriminator works on assessing whether these synthesized examples are fake or not. It works as an inspector that tries not to be fooled by the forger (the generator). The generator synthesizes fake examples using only random noise and the feedback of the discriminator works on improving its quality over time. The two networks keep competing with each other and training in an adversarial way until the generator becomes a master forger that synthesize examples that are very close to the real ones. Consequently, the discriminator cannot detect if these synthesized examples are fake or real. At this stage, the training process is terminated, and the generator model can be saved for later use in testing phase.

There are two different types of GANs; unconditional and conditional (Mirza & Osindero, 2014). Figure 3 shows the difference between both types. The generator of the conditional one has a random noise in addition to a control signal $Y$ that can be a class label, image, video, or text that acts as a condition for the generator to synthesize observations for a certain class or map them from one domain into another.

**Fig. 4:**
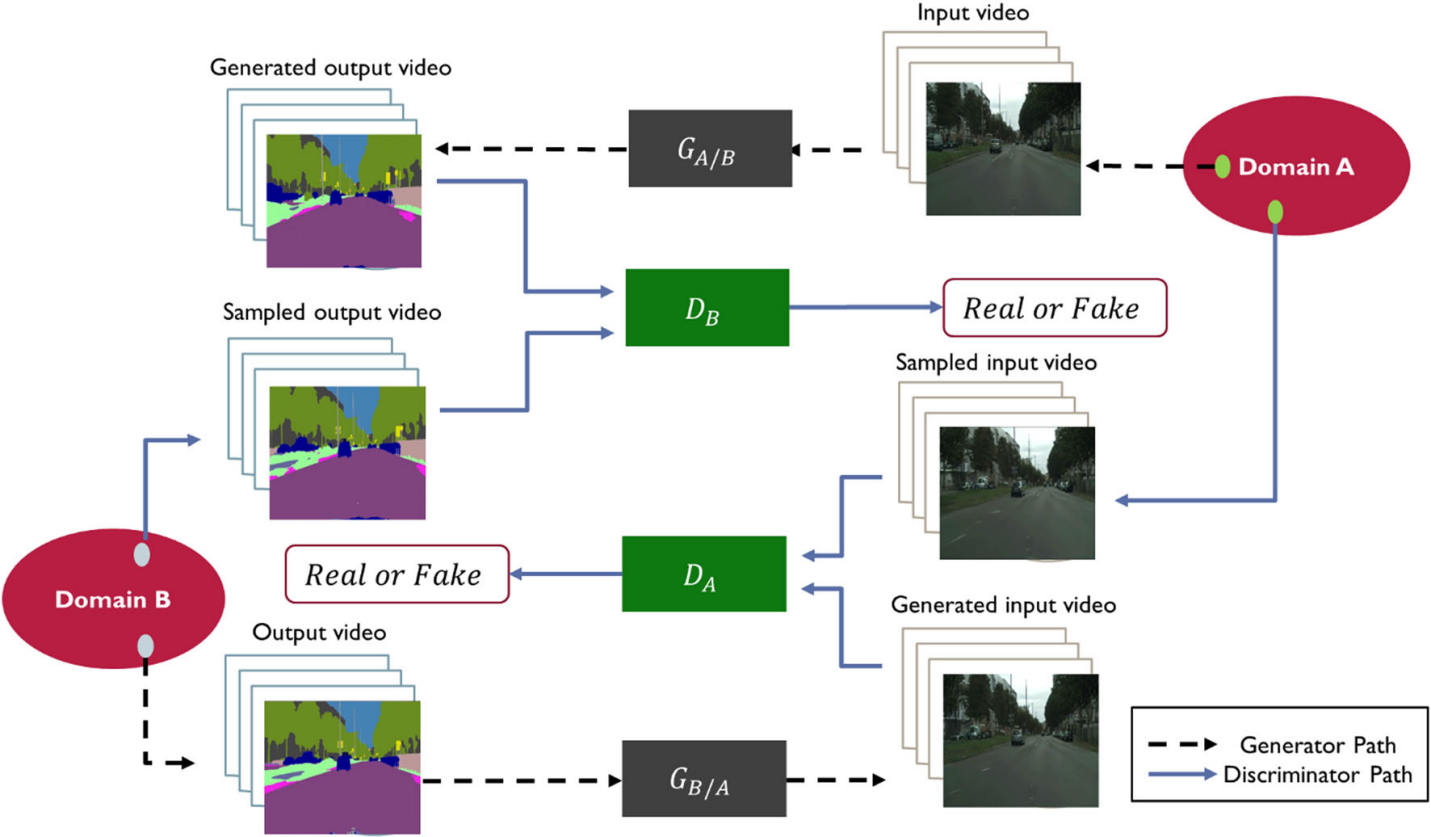
A schematic diagram of the 3D-CycleGAN to translate camera videos into segmented videos (Adopted from Bashkirova et al. (2018))

The conditional GANs (cGANs) are used for image-to-image translation, where an image from a certain domain is mapped into another image in different domain (Elhefnawy et al., 2022; Park et al., 2019). Image translation can be done using cGANs in a supervised or unsupervised way. As a supervised image translation, the pix2pix architecture is proposed in Isola et al. (2017), where the PatchGAN is used to discriminate each local batch of the image instead of the whole image. Another technique that tries to synthesize multiple outputs using the same input is proposed in Zhu et al. (2017b). Other techniques were proposed in X. Liu et al. (2019), Tang et al. (2020), Wang et al. (2018), Zheng et al. (2020) to improve the quality of these supervised image translation approaches. As an unsupervised technique, the CycleGAN is proposed in Zhu et al. (2017a) by adding a cycle consistency loss to enforce an image to be translated from one domain to another domain and translated back into the original domain. Unsupervised video generation techniques are discussed in Srivastava et al. (2015), Vondrick et al. (2016), however none of them considered generating video conditioned on another video. This was tackled in Bashkirova et al. (2018) where the CycleGAN is adapted to 3D-CycleGAN using 3D convolutional layer. The 3D-CycleGAN is depicted in Fig. 4 (adopted from Bashkirova et al. (2018)).

The main idea of the cycle consistency can be formulated mathematically in Eq. 3. The objective of the model is to minimize the adversarial loss (${L}_{GAN}$) of the two generators and discriminators shown in Fig. 4 and the cycle consistency (${L}_{cyc}$) loss for observations $X$ in domain $A$ and observations $Y$ in domain $B$ as defined in Eqs. (4), (5), (6) & (7). See Bashkirova et al. (2018) for more details.

3 $${G}_{A/B}\left({G}_{B/A}\left(x\right)\right)\approx x$$

4 $$  L_{{GAN}} \left( {D_{B} ,G_{{A/B}} ,X,Y} \right) = {\text{E}}_{{y\sim p_{B} }} {\text{log}}\left( {D_{B} \left( y \right)} \right) + {\text{E}}_{{x\sim p_{A} }} {\text{log}}\left( {1 - D_{B} \left( {G_{{A/B}} (x)} \right)} \right) $$

5 $$  L_{{GAN}} \left( {D_{A} ,G_{{B/A}} ,Y,X} \right) = {\text{E}}_{{x\sim p_{A} }} {\text{log}}\left( {D_{A} \left( y \right)} \right) + {\text{E}}_{{y\sim p_{B} }} {\text{log}}\left( {1 - D_{A} \left( {G_{{B/A}} (x)} \right)} \right) $$

6 $${L}_{cyc}({G}_{A/B},{G}_{B/A})={\mathrm{\rm E}}_{x\sim {p}_{A}}\left({\Vert {G}_{A/B}\left({G}_{B/A}\left(x\right)\right)-x\Vert }_{1}\right)+{\mathrm{\rm E}}_{y\sim {p}_{B}}\left({\Vert {G}_{B/A}\left({G}_{A/B}\left(y\right)\right)-y\Vert }_{1}\right)$$

7 $$\begin{aligned}\mathcal{L}\left({G}_{A/B},{G}_{B/A},{D}_{A},{D}_{B}\right)&= {L}_{GAN}\left({D}_{B},{G}_{A/B},X,Y\right)\\ &+ {L}_{GAN}\left({D}_{A},{G}_{B/A},Y,X\right) \\ & + {L}_{cyc}({G}_{A/B},{G}_{B/A})\end{aligned}$$

The generator ${G}_{A/B}$ aims to translating videos from domain $A$ to domain $B$, while the generator ${G}_{B/A}$ translates the videos from domain $B$ to domain $A$. The discriminators ${D}_{B}$ and ${D}_{A}$ can figure out if the translated video fake or real compared to the videos sampled from domain $B$ and domain $A,$ respectively.

Given the successful application of 3D-CycleGAN in unsupervised video-to-video translation, this paper proposes combining the 3D-CycleGAN and polygon generation to solve the problem of time-series prediction. The details of the proposed method are presented in the next section.

## Proposed method for time-series prediction

**Fig. 5:**
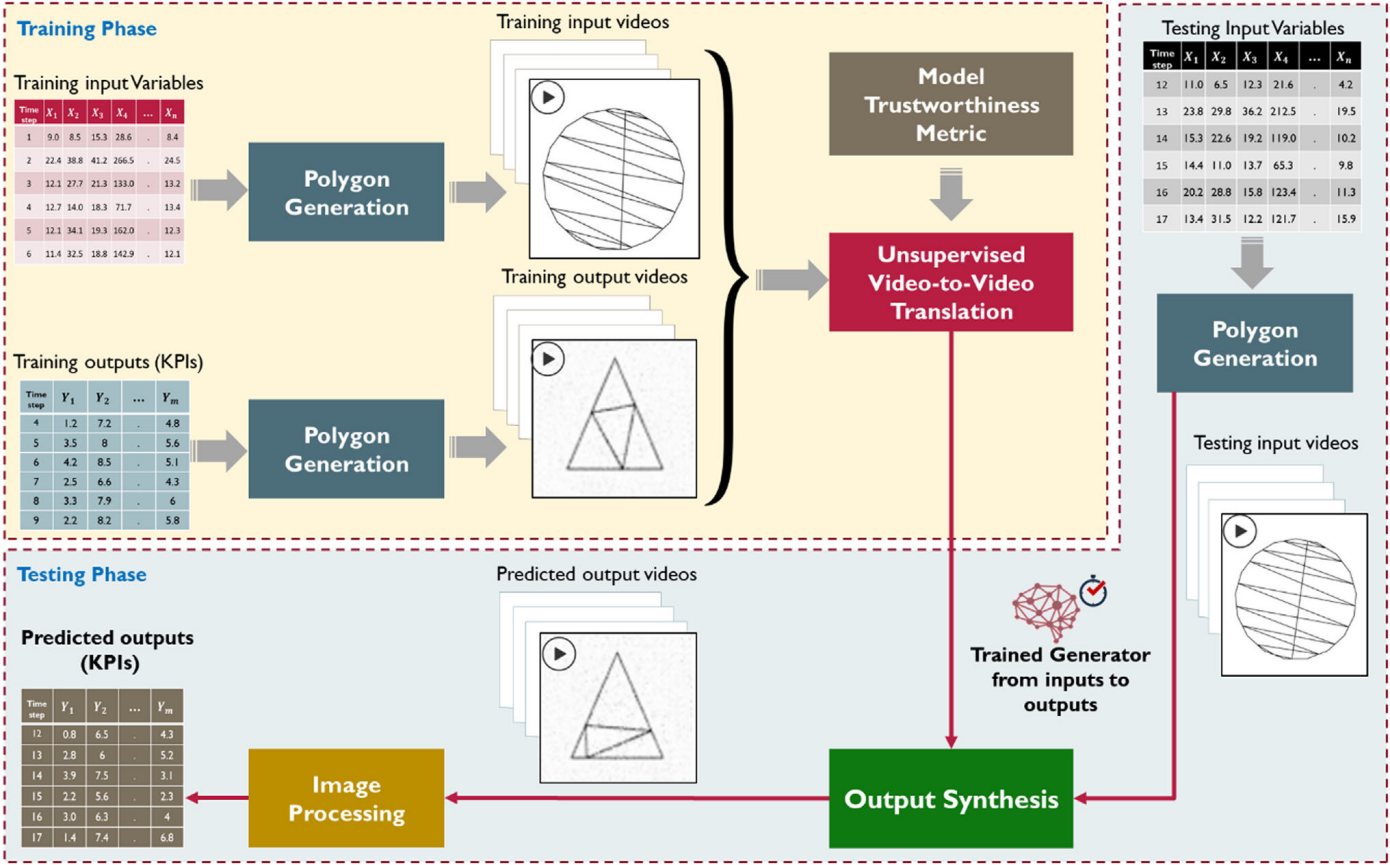
A schematic diagram of the proposed method

The proposed method is comprised of two phases; training and testing, as shown in Fig. 5. The training phase results in a trained generator using the video-to-video translation technique. The generator maps the polygon streams (videos) representing the time-series inputs into other polygon streams representing the outputs (KPIs). In the testing phase, the trained generator translates the input polygon streams (that have not seen before) into other streams representing the predicted outputs. The two phases are illustrated in detail in the following subsections.

### Training phase: unsupervised video-to-video translation

Our proposed method is targeting time-series numeric data with several inputs and outputs. It works in an unsupervised way, where the input variables do not need to be paired with the corresponding outputs. The purpose of this phase is trying to approximate the true distribution of each of the time-series inputs and outputs. As shown in the schematic diagram of the proposed method (Fig. 5), the training time-series data is composed of $n$ numerical input variables (${X}_{1},{X}_{2},\dots , {X}_{n}$) and $m$ numerical outputs (${Y}_{1},{Y}_{2},\dots , {Y}_{m}$). The first step is applying the polygon generation technique for each of the inputs and outputs separately. This results in streams of polygon images (polygon videos) that represent each of the inputs and outputs, as illustrated in “Polygon generation for data representation” section. These streams represent all interrelationships between each of the inputs and outputs in addition to reflecting their changes over time.

**Fig. 6:**
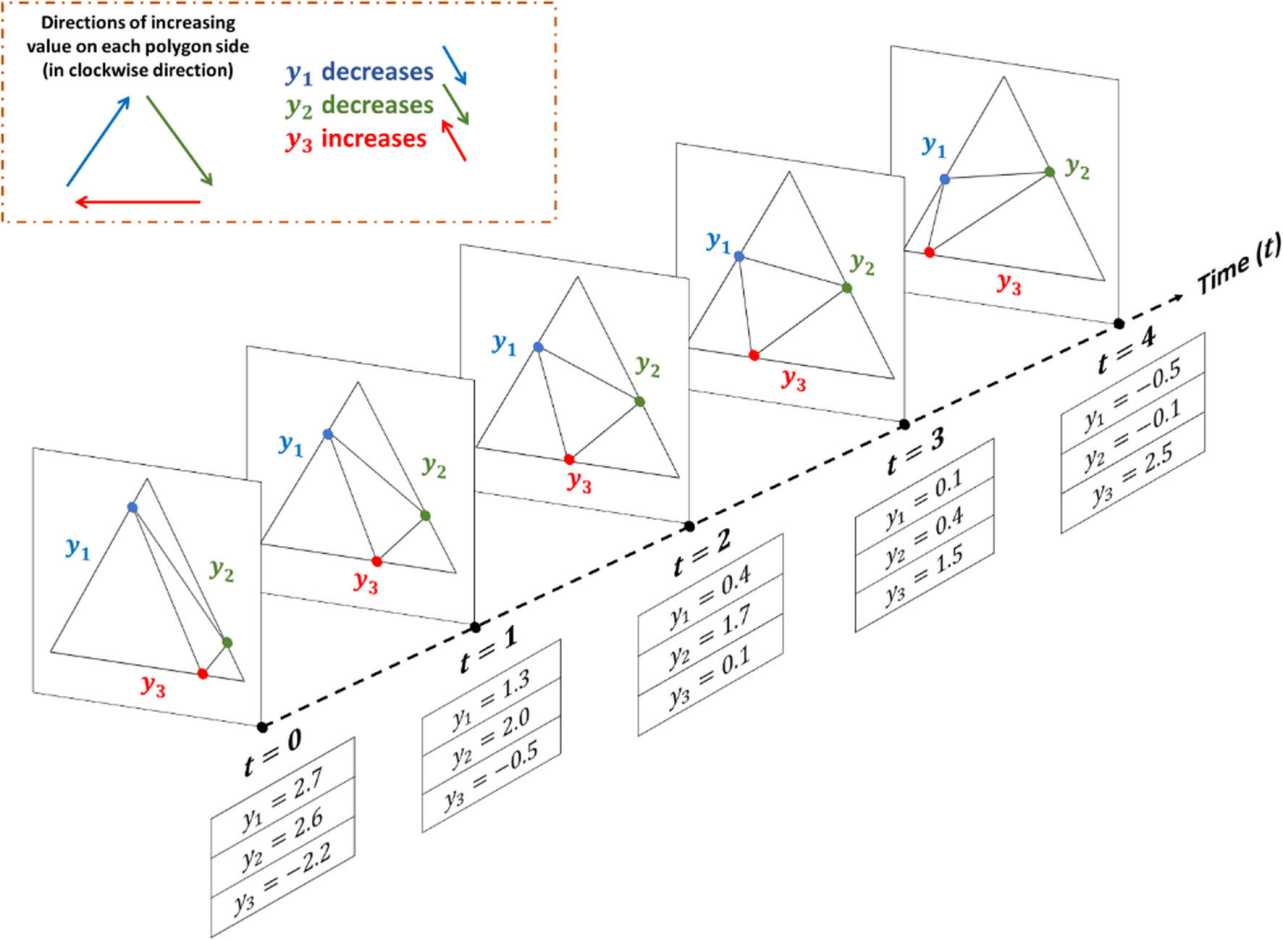
The change of polygon images over time in the form of a polygon stream through an example of three outputs

Figure 6 shows how a polygon changes over time for a data of three outputs (KPIs). For the sake of illustration, the KPIs shown in the figure change monotonically, however, this method can deal with any type of data with changing distribution. As shown in the figure, the movement of the point along the polygon side indicates whether its value increases or decreases over time. In our proposed method, we deal with these polygon videos to capture the true distribution of the input variables and outputs and how to match between them using video-to-video translation technique that is illustrated in what follows.

The unsupervised video-to-video translation technique is fed with both the polygon streams of inputs and outputs. The generator is trained to map the input distribution into the output one. In order to optimize the performance of the video-to-video translation, a model trustworthiness metric is used for ensuring the quality of the synthesized videos compared to the original ones. There are several common metrics for measuring the quality of the video frames such as mean-squared error, peak signal-to-noise ratio, and universal image quality index (Wang & Bovik, 2002). In this work, the universal image quality index proposed in Wang & Bovik (2002) is adopted due to its effectiveness. It adequately compiles the similarity between two videos in terms of different aspects; the correlation, the average of the pixel values and contrast. The quality index is mathematically defined as the multiplication of three terms as defined in Eq. (8):

8 $$Q=\frac{4{\sigma }_{{\varvec{x}}{\varvec{y}}}\overline{{\varvec{x}} }\overline{{\varvec{y}}} }{\left({{\sigma }_{{\varvec{x}}}}^{2}+{{\sigma }_{{\varvec{y}}}}^{2}\right)\left({\left(\overline{{\varvec{x}} }\right)}^{2}+{\left(\overline{{\varvec{y}} }\right)}^{2}\right)}= \frac{{\sigma }_{{\varvec{x}}{\varvec{y}}}}{{\sigma }_{{\varvec{x}}}{\sigma }_{{\varvec{y}}}} . \frac{2\overline{{\varvec{x}} }\overline{{\varvec{y}}} }{{\left(\overline{{\varvec{x}} }\right)}^{2}+{\left(\overline{{\varvec{y}} }\right)}^{2}} . \frac{2{\sigma }_{{\varvec{x}}}{\sigma }_{{\varvec{y}}}}{{{\sigma }_{{\varvec{x}}}}^{2}+{{\sigma }_{{\varvec{y}}}}^{2}}$$

where

$$\overline{{\varvec{x}} }=\frac{1}{N}\sum_{i=1}^{N}{x}_{i},$$

$$\overline{{\varvec{y}} }=\frac{1}{N}\sum_{i=1}^{N}{y}_{i}$$

$${{\sigma }_{{\varvec{x}}}}^{2}= \frac{1}{N-1}\sum_{i=1}^{N}{({x}_{i}-\overline{{\varvec{x}} })}^{2} , {{\sigma }_{{\varvec{y}}}}^{2}= \frac{1}{N-1}\sum_{i=1}^{N}{({y}_{i}-\overline{{\varvec{y}} })}^{2}, {\sigma }_{{\varvec{x}}{\varvec{y}}}= \frac{1}{N-1}\sum_{i=1}^{N}({x}_{i}-\overline{{\varvec{x}} })({y}_{i}-\overline{{\varvec{y}} })$$

where ${\varvec{x}}=\left\{{x}_{i}, i=\mathrm{1,2},\dots ,N\right\}$ represent the polygon stream of the outputs with $N$ frames using polygon generation technique, while ${\varvec{y}}=\left\{{y}_{i}, i=\mathrm{1,2},\dots ,N\right\}$ represent the synthesized polygon stream using our proposed method. The first term of $Q$ ($\frac{{\sigma }_{{\varvec{x}}{\varvec{y}}}}{{\sigma }_{{\varvec{x}}}{\sigma }_{{\varvec{y}}}}$) in Eq. (8) represents the correlation between the frames of the two videos (ranges from -1 to 1), the second term ($\frac{2\overline{{\varvec{x}} }\overline{{\varvec{y}}} }{{\left(\overline{{\varvec{x}} }\right)}^{2}+{\left(\overline{{\varvec{y}} }\right)}^{2}}$) represents how close the mean pixel values of the video frames are (ranges from 0 to 1) and the last term ($\frac{2{\sigma }_{{\varvec{x}}}{\sigma }_{{\varvec{y}}}}{{{\sigma }_{{\varvec{x}}}}^{2}+{{\sigma }_{{\varvec{y}}}}^{2}}$) represents how close the video contrasts are (ranges from 0 to 1).

**Fig. 7:**
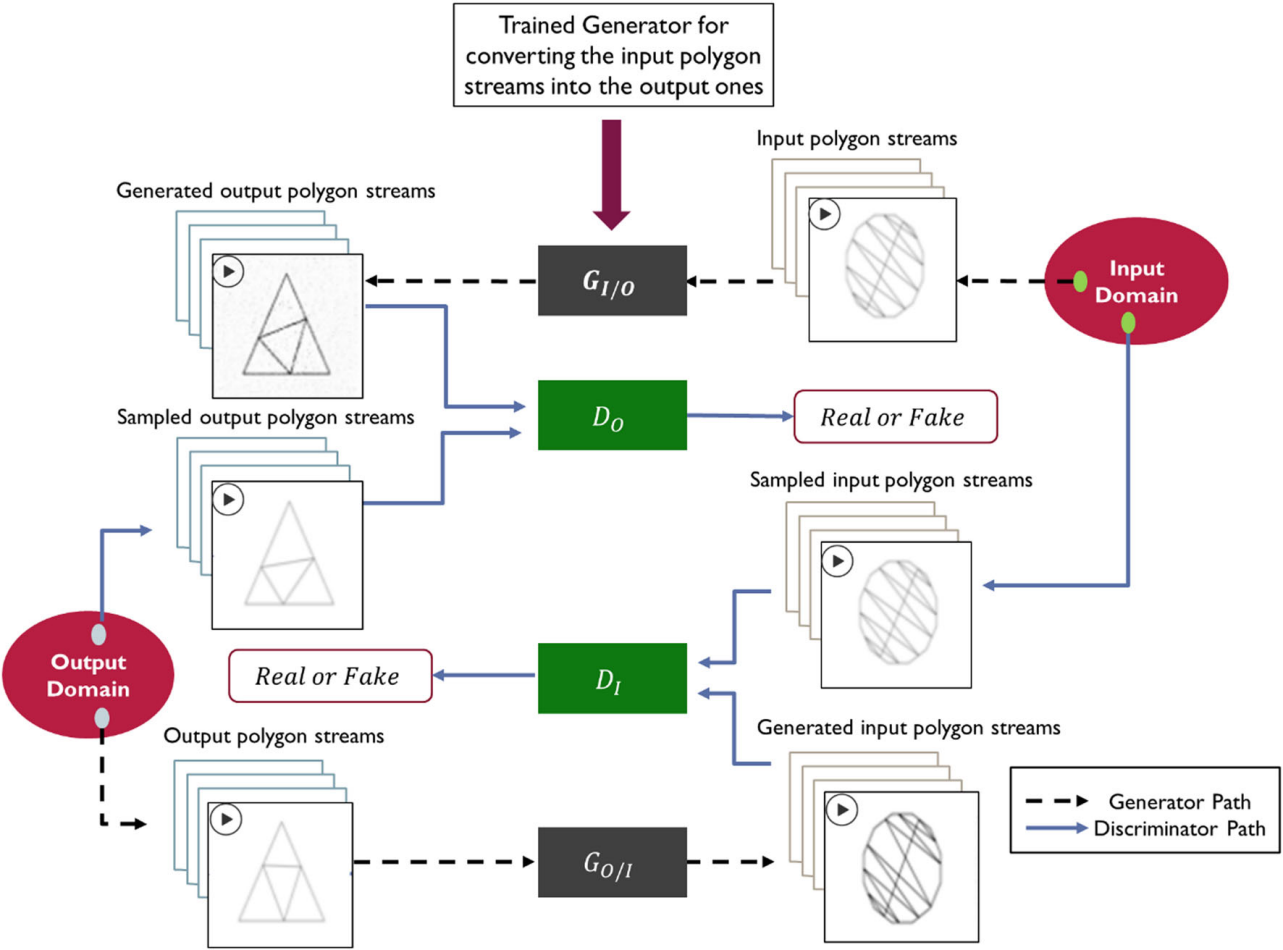
Training of the 3D-CycleGAN model to obtain the generator ${G}_{I/O}$ that converts polygon streams representing time-series input variables into another set of polygon streams representing the time-series outputs

Accordingly, in the proposed method, the video-to-video translation model is tuned based on this quality index metric along with its validation performance to increase the model trustworthiness. Figure 7 illustrates the process of training the unsupervised video-to-video translation using the input and output polygon streams.

The 3D-CycleGAN architecture (Bashkirova et al., 2018) is used for this translation task, where ${G}_{I/O}$ maps the input polygon streams into the output ones, ${G}_{O/I}$ maps the output polygon streams into the input ones. The discriminators ${D}_{I}$ and ${D}_{O}$ differentiate between the real and fake input and output streams, respectively. The ${G}_{I/O}$ is the outcome of the 3D-CycleGAN training phase that is used later in the testing phase.

### Testing phase: mapping videos into time-series outputs

**Fig. 8:**
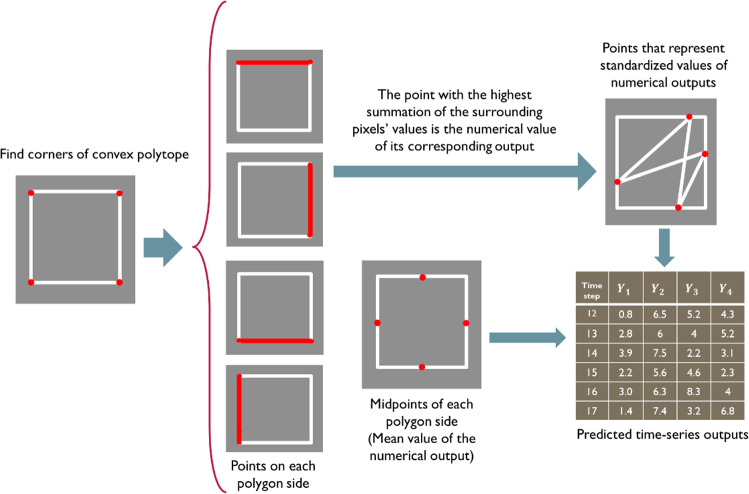
A schematic diagram summarizes the image processing steps for mapping every frame in polygon streams into time-series outputs

In the testing phase, the polygon generation technique is applied for the testing data streams to generate a set of polygon streams representing the testing input variables. The trained generator ${G}_{I/O}$ is used for translating these polygon streams into another set of polygon streams representing the predicted outputs. In order to map the translated streams into predicted outputs, an image processing procedure is applied to every frame in the polygon streams as depicted in Fig. 8 that shows a square representing data with four outputs.

As shown in the figure, first, the corners of the polygon are obtained using the cMinMax algorithm proposed in Chamzas et al. (2020). Based on these corners, the points on each side are determined, where they represent all possible output values. The pixel values in each frame of the polygon stream are binarized, then the point with the highest values of the surrounding pixels represents the standardized numerical value of its corresponding time-series output. Finally, the midpoints of each polygon side (representing the mean values of the outputs) and the points that represent the standardized output values are used to map the polygon streams back to numerical values.

## Case study: concentrator in heat recovery network (HRN)

The proposed method is validated based on a challenging dataset with complex data distribution collected from the concentrator; a major energy-intensive equipment in heat recovery network (HRN) of a pulp and paper mill located in Canada. Details on this equipment, its operation, explanation of the KPIs and the dataset description are discussed in this section.

### System operation and KPIs

In the Kraft pulping process, weak black liquor (BL) is a by-product of wood chips cooking and pulp washing steps (Bajpai, 2018; Biermann, 1996). This weak BL is concentrated in multi effect evaporator and concentrators to increase its solid concentration before to feed the recovery boiler. The objective is to recover the BL inorganics and to burn the organic components. The generated steam in the recovery boiler is used for power generation and for process heating. In order to improve the recovery boiler operation and efficiency, the black liquor solid concentration should be maximized. Typically, multiple-effect evaporation system is used to increase the dissolved solid concentration of the weak BL from 15 to 18% to about 55% and then concentrators are used to concentrate the BL to about 65–70% before entering the recovery boiler.

**Fig. 9:**
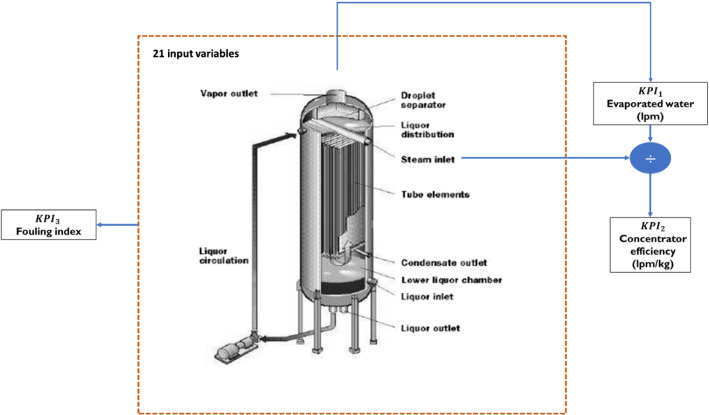
A schematic diagram of the concentrator equipment in the HRN

Figure 9 shows a simplified schematic of the concentrator equipment with the monitored KPIs. The main components of the equipment are a heat exchanger and a flash chamber where vapor is formed and separated from liquid phase (Soualhi et al., 2021). The fresh steam is then used to heat the black liquor in the heat exchanger. More details about the operation of the concentrator are found in Bajpai (2018).

As shown in Fig. 9, the first KPI is the evaporated water flow, the second is the concentrator efficiency where it is calculated as the evaporated water divided by the fresh steam consumed. The third KPI is the fouling index which is an important indicator of the decrease in the overall heat transfer from steam to black liquor. Since the heat exchange rate depends on the temperature difference between the steam and the black liquor, the fouling index is defined using Eq. (9) (Ardsomang et al., 2013).

9 $$Fouling\, index =\frac{temperature\, of \,steam - temperature\, of\, heavy\, black \,liquor}{evaporated\, water}$$

### Experimental setup

The concentrator dataset composed of 37,440 observations collected from the mill historian of 390 days with sampling time of 15 min. It comprises a total of 42 cycles and includes a total of 21 manipulated and measured variables; selected by the process expert to represent the highly dynamic behavior of the concentrator operation. Examples of these variables are shown in Table 3. Data cleaning and preparation through removal of outliers and non-representative data were done by the process expert using the software EXPLORE (Amazouz, 2015).

**Table 3:** Examples of manipulated, measured variables and KPIs for the concentrator equipment

Manipulated and measured variables	KPIs
• Liquor flow to concentrator (lpm) • Temperature of liquor from concentrator (°C) • Temperature of vapor from concentrator (°C) • Pressure of fresh steam to concentrator (kPa) • Fresh steam flow to concentrator (kg/h) • Temperature differential steam/liquor concentrator (°C) • Temperature differential steam/liquor concentrator (°C)	• ${KPI}_{1}$: Evaporated water (lpm) • ${KPI}_{2}$: Concentrator efficiency (evaporated water (lpm) / steam (kg)) • ${KPI}_{3}$: Fouling index

**Fig. 10:**
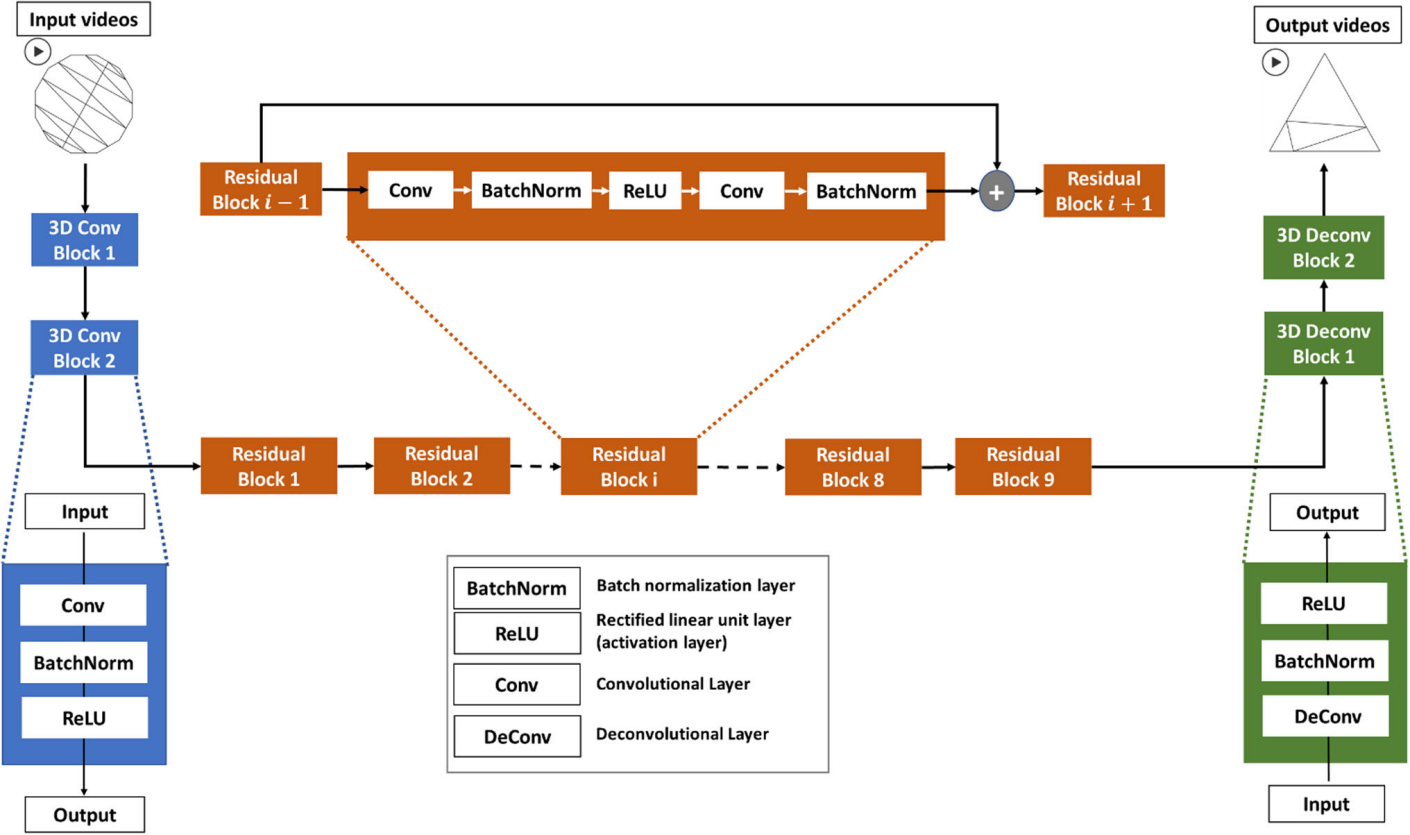
The generator architecture in the 3D-CycleGAN

In this work, we used the 3D-CycleGAN proposed in Bashkirova et al. (2018) with two generators and two discriminators. The generator architecture (Johnson et al., 2016) is illustrated in Fig. 10, where it is composed of two 3D convolutional blocks followed by nine residual blocks and two 3D deconvolutional blocks for upsampling. Each convolutional block is composed of a 3D convolutional layer (Ji et al., 2012), batch normalization layer and rectified linear-unit (ReLU) as an activation layer. Each deconvolutional block is composed of 3D deconvolutional layer, batch normalization layer and ReLU layer. The residual block is composed of five layers ordered as follows: 3D convolutional, batch normalization, ReLU, 3D convolutional and batch normalization. The output of each residual block is added to that of the previous block as input to the next residual block as shown in Fig. 10. Since deep neural networks often suffer from the vanishing gradient and performance degradation, the residual block is used to mitigate this effect (He et al., 2016).

**Fig. 11:**
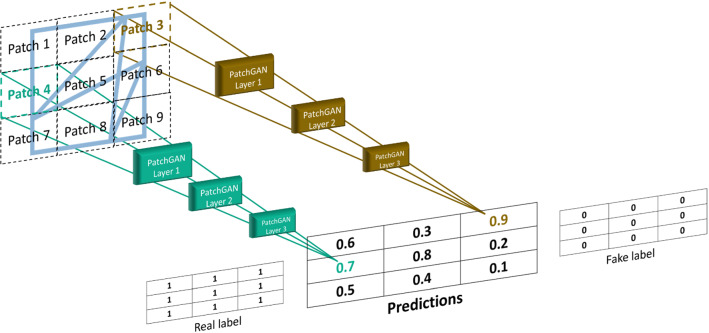
- PatchGAN: The discriminator of the 3D-CycleGAN

The discriminator in the 3D-CycleGAN is the PatchGAN architecture introduced in Demir and Unal (2018). PatchGAN divides each video into 70 * 70 * *h* patches, where *h* is the video depth. This architecture predicts the classification probability in a form of a 3D matrix where every value refers to the probability for the corresponding patch in the video frame. For the sake of simplification, we use a single image as a video frame in Fig. 11 to illustrate the operation of PatchGAN. The 3D matrix with all entries of ones refers to a real video, while the one with zeros refers to a fake one.

The TensorFlow (Abadi et al., 2016) with Python 3.7 was used to implement, train and test the proposed method (PG + 3D-CycleGAN) and other baseline algorithms on a high performance computing (HPC) infrastructure in Natural Resources Canada with following specifications: Intel® Xeon® Gold 6140 CPU @2.3 GHz, 1 TB of RAM + 4 GPUs (NVIDIA Tesla V100).

## Results, discussion & future work

The performance of the proposed method is compared with other baseline time-series predictors; recurrent neural network (RNN), long-short term memory (LSTM), one-dimensional convolutional neural network (1D-CNN). These time-series predictors have been used extensively in the literature and in practice (Dong et al., 2017; Lanzetti et al., 2019; Soualhi et al., 2021; Zagrebina et al., 2019). The hyperparameters for each baseline predictor are optimized using grid search for the sake of the best performance. The tuning of our proposed method was based on the model trustworthiness metric (universal image quality index) that mentioned in “Proposed method for time-series prediction” section. The goal is to maximize the value of this index to ensure that the synthesized videos are structurally close to the desired videos that represent the time-series outputs.

It is worth mentioning that, during the process of training the 3D-CycleGAN using polygon streams, there was a problem of generating the same output video for multiple input videos. This phenomenon is common in GAN training, and it is called “mode-collapse” (Durall, Chatzimichailidis, Labus, & Keuper, 2020). The real distribution of the time-series outputs in most industrial processes is a multi-modal distribution due to the highly nonlinear dynamic nature of these systems. The generator sometimes can fool the discriminator through synthesizing fake videos with only one mode, while the discriminator cannot figure out if it is fake or not. There are some hacks to overcome this problem such as the normalization of input videos: the grey-scale videos can be normalized to have the values in interval [-1,1] or [0,1] instead of [0,255]. Another hack is to decrease the learning rate of the optimizer used in generator and discriminator. After we followed these two hacks in this case study, the 3D-CycleGAN was able to synthesize multi-modal output videos.

Table 4 shows the range of the hyperparameters for grid search in each time-series predictor. A random seed is fixed for the reproducibility of the results. Both R-squared values (${R}^{2}$) and root mean square error ($RMSE$) are used as metrics to compare the performance of all predictors (Bustillo et al., 2020; Kasuya, 2019).

**Table 4:** Range of hyperparameters of each time-series predictor using the concentrator equipment

Algorithm	Hyperparameters
Proposed method (PG + 3DCycleGAN)	# filters in conv layer = [4,64], filter size = (2,3) # epochs = [30,150]
RNN & LSTM	# units = [15,40], activation function = {sigmoid, ReLU, tanh}, recurrent activation = {sigmoid, ReLU, tanh}, dropout = [0,1]
1D-CNN	# filters = [4,32], batch size = [4,32], # epochs = [10,50], activation function = {sigmoid, ReLU}, kernel size = [2.8]

Moreover, a penalty function is used as a validation criterion for each time-series predictor, taking into consideration the underestimation and overestimation of the three KPIs. The penalty function ${L}^{j}({t}_{k})$ of ${KPI}_{j} , j=\mathrm{1,2},3$ at instance ${t}_{k}$ is defined in Eq. (10).

10 $${L}^{j}({t}_{k})= \left\{\begin{array}{l}{\alpha }_{j}u\left(j\right)\left({KPI}_{j}\left({t}_{k}\right)-\widehat{{KPI}_{j}}\left({t}_{k}\right)\right) , \widehat{{KPI}_{j}}\left({t}_{k}\right)u(j)< {KPI}_{j}\left({t}_{k}\right)u(j) \\ 0 , \widehat{{KPI}_{j}}\left({t}_{k}\right)= {KPI}_{j}\left({t}_{k}\right) \\ {\beta }_{j}u(j)\left(\widehat{{KPI}_{j}}\left({t}_{k}\right)-{KPI}_{j}\left({t}_{k}\right)\right) , \widehat{{KPI}_{j}}\left({t}_{k}\right)u(j)> {KPI}_{j}\left({t}_{k}\right)u(j)\end{array} \right.$$

where ${\alpha }_{j}$ and ${\beta }_{j}$ are the underestimation and overestimation parameters for each ${KPI}_{j},$ respectively and $\widehat{{KPI}_{j}}\left({t}_{k}\right)$ and ${KPI}_{j}\left({t}_{k}\right)$ are the predicted and true values of ${KPI}_{j}$ at instance ${t}_{k},$ respectively. The term $u(j)$ has a value of 1 or -1 depending on the predicted ${KPI}_{j}$. $u\left(1\right)=u\left(2\right)=1$ (the overestimation of ${KPI}_{1}$ and ${KPI}_{2}$ is penalized more than the underestimation), while $u\left(3\right)= -1$ (the underestimation of ${KPI}_{3}$ is penalized more than the overestimation). These parameters were assigned according to the energy efficiency importance of each KPI as confirmed by the process expert. Accrodingly, in this work, the values of ${\alpha }_{j}$ and ${\beta }_{j}$ are assigned the values shown in Table 5.

**Table 5:** Underestimation $({\alpha }_{j})$ and overestimation $({\beta }_{j})$ parameters for each ${KPI}_{j}$ in the penalty function as defined by the process expert

	$j=1$	$j=2$	$j=3$
${\alpha }_{j}$	0.1	0.1	0.1
${\beta }_{j}$	0.15	0.3	0.2

The average penalty score for each KPI is calculated as shown in Eq. (11).

11 $${L}_{AP}^{j}= \frac{1}{N}{\sum }_{k=1}^{N}{L}^{j}({t}_{k}),$$

where $N$ is the total number of time steps. The total average penalty score for each time-series regression model is calculated as shown in Eq. (12).

12 $${L}_{model}= \frac{1}{3}{\sum }_{j=1}^{3}{L}_{AP}^{j}.$$

### Results

As previously mentioned in “Case study: concentrator in heat recovery network (HRN)” section, three KPIs are used for this case study; the evaporated water flow, the concentrator efficiency, and the fouling index. Based on the polygon generation technique and the number of input variables in concentrator data (21 variables), each observation has 10 different Hamiltonian cycle connections (10 polygon streams). These streams represent all interrelationships between the input variables and their changes over time, while there is only one Hamiltonian cycle connection (one polygon stream) for the 3 outputs. It is worth mentioning that the choice of the number of frames per video is limited by the memory of a single GPU unit. Therefore, the number of frames is set to 30 per video using the HPC infrastructure mentioned previously.

The R-squared and $RMSE$ values of all predictors are listed in Table 6 and the total average penalty incurred from the erroneous prediction of each predictor is listed in Table 7 for the concentrator case study. As shown in Tables 6 and 7, the proposed method (PG + 3D-CycleGAN) has achieved the highest R-squared value and lowest $RMSE$ on each of the KPIs and the lowest total average penalty score. The numbers in bold indicate the best results obtained. It can be observed from the results that there is a significant improvement of the prediction of the concentrator efficiency (${KPI}_{2}$).

**Table 6:** R-squared and root mean square error values of each algorithm in the concentrator dataset

Algorithm	${KPI}_{1}$		${KPI}_{2}$		${KPI}_{3}$	
	${R}^{2}$	$RMSE$	${R}^{2}$	$RMSE$	${R}^{2}$	$RMSE$
Proposed Method (PG + 3D CycleGAN)	0.75	14.63	0.8	0.014	0.95	0.029
RNN	0.64	24.6	0.63	0.033	0.79	0.062
LSTM	0.69	18.44	0.63	0.026	0.93	0.038
1D-CNN	0.67	19.58	0.62	0.029	0.92	0.037

**Table 7:** Total average penalty scores for each algorithm in the concentrator dataset

Algorithm	PG + 3D CycleGAN	RNN	LSTM	1D-CNN
Total average penalty score	**0.0261**	0.0315	0.0294	0.0309

**Fig. 12:**
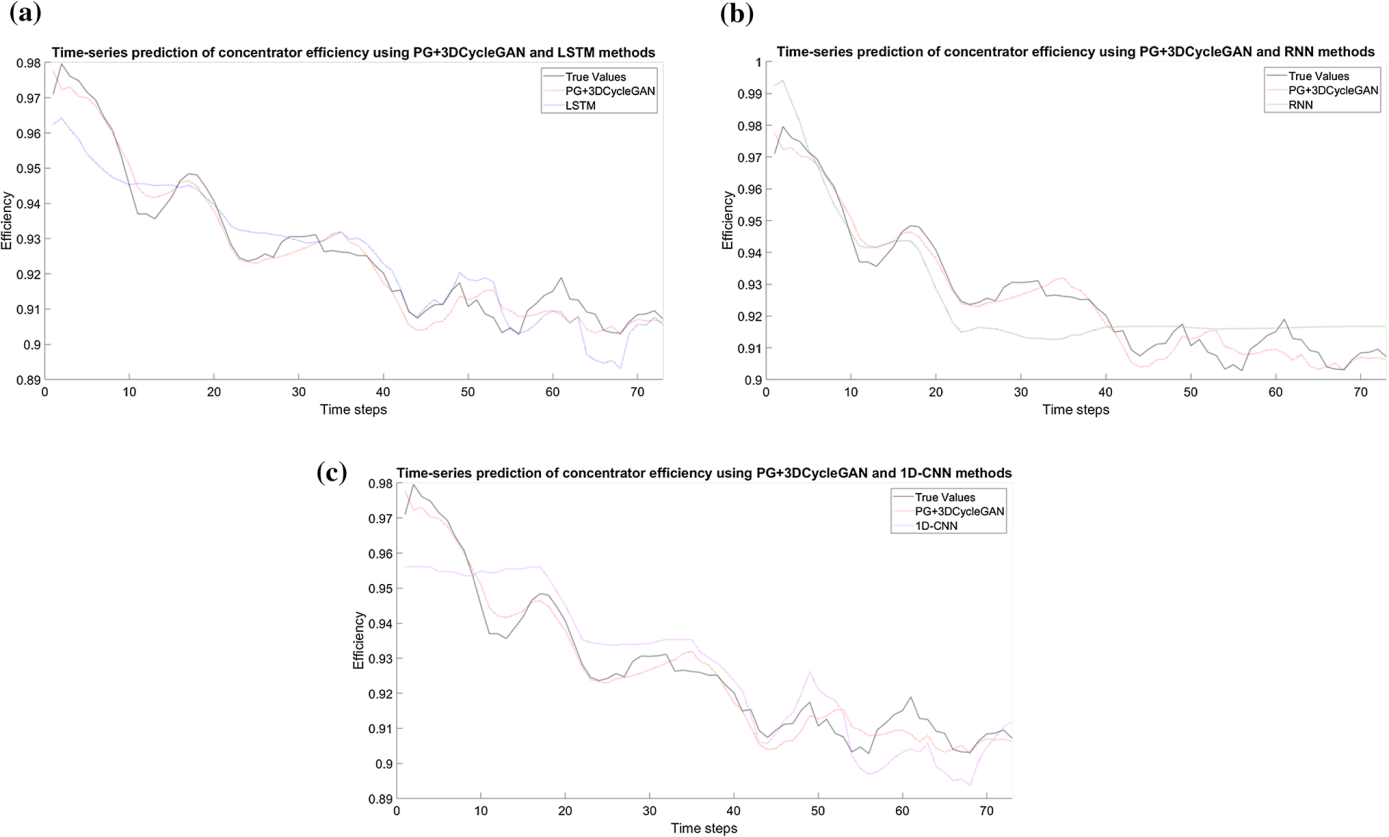
Prediction of ${KPI}_{2}$ (concentrator efficiency) using the proposed method (PG + 3DCycleGAN) and other prediction models **a** LSTM **b** RNN **c** 1D-CNN

Figure 12 visualizes the performance of the time-series prediction of the proposed method in comparison with the true values and every baseline prediction model. It shows the predicted values of the concentrator efficiency over time.

These results are validated by the process expert and shown to be useful for the mill operator. This helped better monitor such highly dynamic operation and mitigating the economic losses and environmental impacts resulting from the past inaccurate prediction over time. Besides, this helped the operator prescribe the proper actions in real-time.

### Discussion and future work

**Fig. 13:**
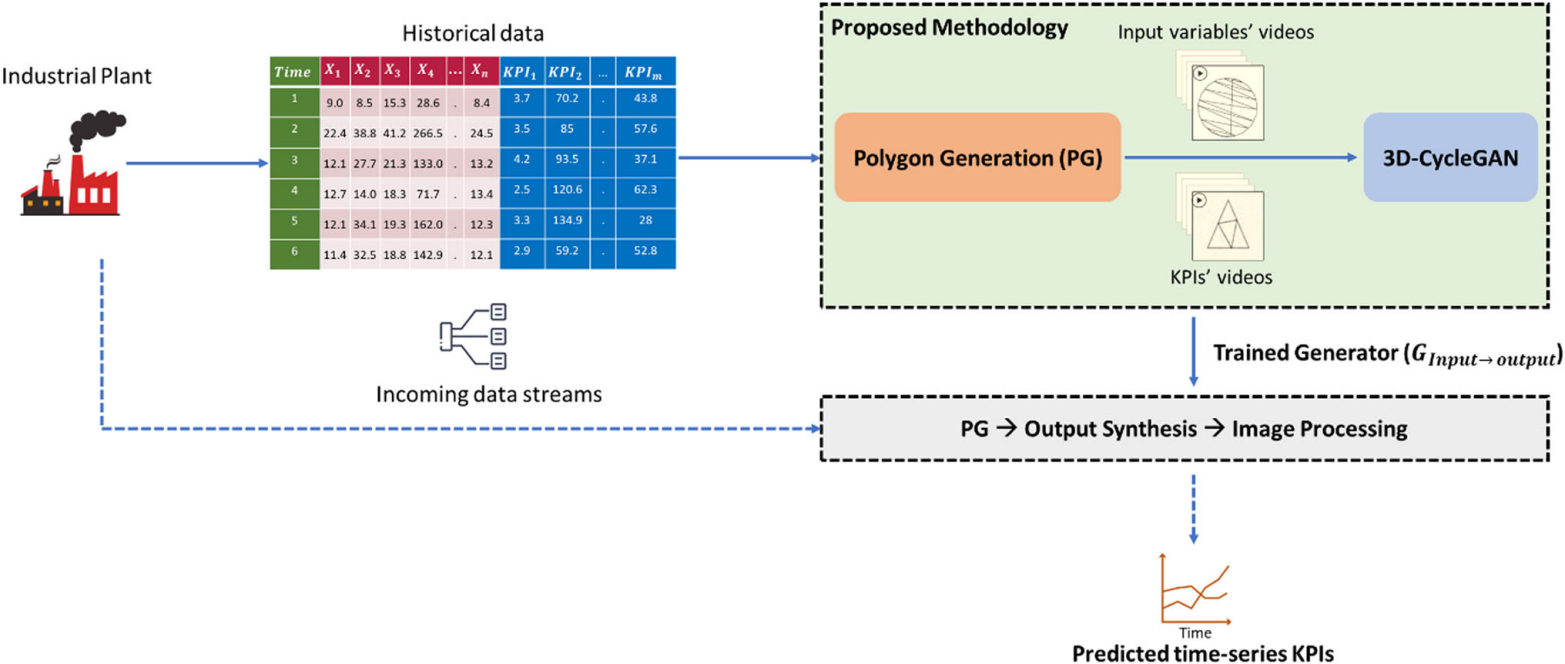
A schematic diagram summarizes the application of PG + 3DCycleGAN methodology for predicting KPIs in industrial processes

To sum up, the historical data is collected from the industrial plant through multiple sensors, accordingly, this time-series data represents the fuel of our proposed method. The proposed method was able to solve the problem of distribution matching of input variables and the outputs in this challenging industrial dataset. This is attributed to the following facts. Both of the time-series input variables and KPIs are converted into polygon videos to train the unsupervised video-to-video translation architecture (3D-CycleGAN) in the training phase. It is worth mentioning that the testing phase is neither computationally expensive nor time consuming. A fast and accurate testing phase is a desired characteristic from an industrial perspective. This testing phase in the proposed method includes preparing polygon videos compiling the data stream of input variables, then testing the trained generator to map these videos to other set of videos that represent the predicted KPIs, finally the numerical KPI values are recovered easily using the image procedure mentioned previously. Figure 13 summarizes the implementation of proposed methodology for predicting KPIs in industrial processes. Most industrial systems have a non-stationary behavior represented by data distributions that are varying over time and are challenging to be modeled. In most industrial systems, the KPIs and the system variables are defined by the process expert prior to the modeling stage. Our proposed methodology including the PG process is generic and can be adapted to work with systems with changing numbers of variables and KPIs by easily generating new synthesized polygon videos corresponding to the inputs and outputs, respectively.

The polygon generation technique used in the proposed method was able to express all interrelationships between the input variables and the KPIs based on the Hamiltonian cycles. This efficiently represents the numerical data and leverage its quality as one of data-centric AI goals. Moreover, to ensure the consistency of the structure of the translated polygon streams, a model trustworthiness metric is used to tune the architecture of the 3D-CycleGAN to maximize the universal image quality index for each video frame.

Besides the above strengths, we make use of the breakthrough of the DL and its impressive performance in the computer vision problems especially in generative modeling. DL can capture the high dynamic behavior of the equipment with minimal intervention of the process expert. It is worth mentioning that the proposed method is an end-to-end learning process that does not need the effort of manual feature engineering that is done by the process expert in a tedious manner. In addition, it saves the expert’s effort for the labeling process as the method works in an unsupervised way. Besides, the availability of advanced IT infrastructure in modern industries makes the proposed method feasible especially in the training phase, in which training a deep architecture using massive amount of data is needed.

From the practical point of view, all these merits can guarantee the operationally deployable implementation of the proposed method in industrial settings. Other challenging industrial datasets will be collected from a number of non-linear and dynamic processes in the future for further testing of our proposed method. The resolution of the polygon videos will be further investigated as it may have a significant effect on the method performance.

The proposed method opens the door for industrial data fusion in terms of merging numerical data, images, and videos. This can help efficiently exploit the available heterogeneous data to maximize the global value of isolated data silos provide the operator with valuable knowledge. One of our future research directions is to develop a platform that can integrate and process different types of data in terms of structure and format. The platform will consist of an ensemble of DL models each used to process specific data type. Moreover, the visualization of the KPIs changes and the input variables in the form of representational videos can play a key role in interpretation of DL models. The final goal is to provide the end users with accurate and transparent knowledgebase with explainable rules.

## Conclusion

A novel and powerful time-series prediction method based on two main building blocks; polygon generation and unsupervised video-to-video translation is proposed and successfully tested on a real application. The time-series numerical observations are converted into a set of polygon streams (videos) using polygon generation. The unsupervised video-to-video translation is used to map the videos representing the input variables into others representing the outputs as a distribution matching problem. The proposed method takes advantage of the unprecedent performance of generative deep learning (DL) modeling to capture the dynamic and complex data distribution, which is hard to determine in highly non-linear process industries. The method is tested successfully using a challenging industrial dataset collected from a concentrator equipment in a thermomechanical pulp mill located in Canada. The results show that the proposed method outperformed other comparable time-series DL predictors in terms of KPIs’ prediction accuracy. This helped maintain an energy-efficient operation of the mill. This proves that the proposed method has a potential to monitor various complex industrial equipment. As the proposed method has the advantage of working in an unsupervised way, it saves the effort of data labeling process done by the process expert. The trustworthiness of the video translation model is maximized using an index to maintain a consistent structure of the translated polygon streams. Moreover, the interpretability of DL models is one of our current research directions. Besides, using polygon generation as a data representation technique opens the door for fusion of heterogeneous data types in various industrial processes.

## Abbreviations

$${X}_{j}$$: $${j}^{th}$$ Input variable
$${Y}_{h}$$: $${h}^{th}$$ Output
$\widehat{q}, \widehat{l}$: The unit vector of $x$ and $y$ directions respectively
$\overline{{X }_{j}}$: Mean of ${j}^{th}$ input variable
$\overline{{Y }_{h}}$: Mean of ${h}^{th}$ output
${\delta }_{j}$: Standard deviation of ${j}^{th}$ input variable
${\delta }_{h}$: Standard deviation of ${h}^{th}$ output
${x}_{kj}$: Value of ${j}^{th}$ input variable for ${k}^{th}$ observation
${y}_{kh}$: Value of ${h}^{th}$ output for ${k}^{th}$ observation
${Z}_{kj}$: Standardized value of ${j}^{th}$ input variable for ${k}^{th}$ observation
${Z}_{kh}$: Standardized value of ${h}^{th}$ output for ${k}^{th}$ observation
$\widehat{{X}_{j}}$: Unit vector of the polygon side representing ${j}^{th}$ input variable
$\widehat{{Y}_{h}}$: Unit vector of the polygon side representing ${h}^{th}$ output
$\overrightarrow{{X}_{j}}$: Point coordinates of the zero standardized value of the variable ${X}_{j}$
$\overrightarrow{{Y}_{h}}$: Point coordinates of the zero standardized value of the output ${Y}_{h}$
$\overrightarrow{{X}_{j}^{k}}$: Point coordinates of the standardized values of ${k}^{th}$ observation for variable ${X}_{j}$
$\overrightarrow{{Y}_{h}^{k}}$: Point coordinates of the standardized values of ${k}^{th}$ observation for output ${Y}_{h}$
${G}_{B/A}$: Generator of the GAN architecture translating observations from domain $B$ to domain $A$
${G}_{A/B}$: Generator of the GAN architecture translating observations from domain $A$ to domain $B$
${D}_{A}$: Discriminator of the GAN architecture for observations generated from domain $A$
${D}_{B}$: Discriminator of the GAN architecture for observations generated from domain $B$
${L}_{GAN}$: Adversarial loss of the GAN architecture
${L}_{cyc}$: Cycle consistency loss of the GAN architecture
$\mathcal{L}$: Total loss of the GAN architecture
$Q$: Universal image quality index
$\overline{x }$: Mean of the pixel values of the frames of polygon stream representing the outputs
$\overline{y }$: Mean of the pixel values of the frames of synthesized polygon stream using the GAN model
${\sigma }_{x}$: Standard deviation of the pixel values of the frames of polygon stream representing the outputs
${\sigma }_{y}$: Standard deviation of the pixel values of the frames of synthesized polygon stream using the GAN model
${\alpha }_{j}$: Underestimation parameter of ${KPI}_{j}$ in the penalty function
${\beta }_{j}$: Overestimation parameter of ${KPI}_{j}$ in the penalty function
${L}^{j}\left({t}_{k}\right)$: Penalty function of ${KPI}_{j}$ at instance ${t}_{k}$
${L}_{AP}^{j}$: Average penalty function of ${KPI}_{j}$
${L}_{model}$: Total average penalty function of each time-series regression model
